# Introducing Copula as a Novel Statistical Method in Psychological Analysis

**DOI:** 10.3390/ijerph18157972

**Published:** 2021-07-28

**Authors:** Elham Dehghani, Somayeh Hadad Ranjbar, Moharram Atashafrooz, Hossein Negarestani, Amir Mosavi, Levente Kovacs

**Affiliations:** 1 Department of Psychology, Rafsanjan Branch, Islamic Azad University, Rafsanjan 7718184483, Iran; m.e6165@yahoo.com; 2 Department of Theology and General Islamic Courses, Vali-e-Asr University of Rafsanjan, Rafsanjan 7718897111, Iran; s.hadad@vru.ac.ir; 3 Imam Khomeini Specialized Center, Islamic Counseling Faculty, Qom 3713755518, Iran; moharramatashafrooz@gmail.com; 4 Department of Statistics, Faculty of Mathematical Sciences, Vali-e-Asr University of Rafsanjan, Rafsanjan 7718897111, Iran; negarestani@vru.ac.ir; 5 John von Neumann Faculty of Informatics, Obuda University, 1034 Budapest, Hungary; 6 Biomatics Institute, John von Neumann Faculty of Informatics, Obuda University, 1034 Budapest, Hungary; 7 ELKH SZTAKI Institute, P.O. Box 63, 1518 Budapest, Hungary; 8 Physiological Controls Research Center, University Research and Innovation Center, Obuda University, 1034 Budapest, Hungary

**Keywords:** self-esteem, anxiety, psychology, copula, probability matrix, probability theory, statistics, dependence structure, mathematical modeling, social science data

## Abstract

During the past decades, the relationship between various psychological parameters had been studied in detail. However, the dependency structure of correlated parameters was rarely investigated. Knowing the dependence structure helps in finding the probability matrix of the interaction between the parameters. In this research, a novel approach was introduced in psychological analysis using copula functions. For this purpose, the self-esteem and anxiety of 141 university students in Iran were extracted using the Coopersmith Self-esteem Inventory and the Zang Anxiety Scale. Then the dependence structure of self-esteem and anxiety were established using copula functions. The Frank copula achieved the best fit for the joint variables of self-esteem and anxiety. Finally, the probability matrix of different classes of anxiety, taking into account self-esteem classes, was extracted. The results indicated that poor self-esteem leads to severe or very severe anxiety, with more than 98% probability, while strong self-esteem may lead to normal and mild anxiety, with about 80% probability. It can be concluded that the method was promising, and that copula functions can open a window to the dependence structure analysis of psychological parameters.

## 1. Introduction

Anxiety disorder is the third most common type of mental disorder after major depressive disorder and alcohol dependence [1]. Epidemiological studies have shown that the prevalence rate of lifelong anxiety disorder is 13.3% [1]. Over the past few decades, several studies have been conducted on student anxiety [2,3,4,5,6]. These studies have shown that the experience of anxiety during schooling creates problems in the next stages of the students’ lives [7] that, in addition to personal discomfort, may have a negative impact on their job and daily performance [8]. Researchers and clinicians, in recent years, have made increasing efforts to understand the widespread effects of anxiety disorder. Numerous studies have examined the prevalence, etiology, and treatment of this clinical problem [3,9,10]. This shows that anxiety is no longer a forgotten disorder [11]. Quantitative [12] and qualitative [13] research studies have shown that the low self-esteem has an adverse effect on the mental health. On the other hand, improvement and strengthening of the self-esteem would be essential in the process of recovery from mental illness [14]. Research studies have shown that negative self-evaluation, the fear of evaluating others, the avoidance of situations that involve evaluation, and expecting others to evaluate one negatively are among the most obvious characteristics of social phobia [15,16]. In recent years, researchers have cited high self-esteem as an important source of support that people can use as a risk factor in the face of adverse events in daily life [17] in order to reduce their effects. According to the researchers’ view, self-acceptance, which is defined as having a positive attitude towards oneself, is one of the major variables in mental health [18]. Self-esteem depends on a positive attitude towards oneself. The amount of anxiety that people experience in the face of different situations is not due to the situation itself. It is because of the perception and mentality that one has of oneself in those circumstances. This is where self-esteem comes into play. People’s self-esteem creates an image of their abilities in their mind. This mental imagery affects the type and quality of behavior in anxious situations. A person who does not believe in him/herself feels severe helplessness in the face of danger and is sure that he cannot survive in the face of this problem. With such a belief, one has problems even with daily tasks and challenges. Every problem seems bigger than usual and causes anxiety to cover his whole mind and body. The connection between self-esteem and anxiety is not limited to this. A person who suffers from the self-esteem personality disorder usually does not have the ability to resist the demands of others. Such person often accepts different tasks and responsibilities and without paying attention to his or her ability for accomplishment. One of the most important effects that self-esteem has on human beings is the feeling of dignity and avoidance of self-blame and feelings of guilt, and this prevents anxiety. Because there is a correlation between people’s perception of their abilities and their self-esteem, whenever a person has a positive evaluation of his performance, his anxiety decreases and his self-esteem increases, while a negative evaluation of performance increases anxiety and decreases self-esteem [19]. High self-esteem is associated with lower levels of anxiety [20]. In a study of undergraduate students, Izgic et al. (2004) [21] found that the highest prevalence of anxiety was among students with low self-esteem and the lowest prevalence of anxiety was among students with high self-esteem. In a similar study of undergraduate students, Crum and Pratt (2001) [22] showed that the low self-esteem may lead to anxiety.

Bajaj et al. (2016) [23] examined the mediation effect of self-esteem on the relationship between mindfulness and anxiety. Their study explored the effect of mindfulness on the anxiety considering the self-esteem. Mustafa et al. (2015) [24] studied self-esteem and anxiety in university students in Albania and Kosovo. They reported that self-esteem and gender were adversely correlated. Papazisis et al. (2014) [25] investigated the relationship between religious and spiritual beliefs and anxiety. They reported a strong positive correlation between religious and spiritual beliefs and self-esteem. Considering the negative effects of anxiety on students’ academic performance during their studies and on their future careers and lives, the present study was conducted to determine the relationship between self-esteem and anxiety and its components among students. The main question of the study was whether students with different levels of self-esteem experience different levels of anxiety. Based on the findings of previous research, the study hypothesized that students with high self-esteem were likely to experience lower levels of anxiety and its components. In other words, there is an inverse relationship between self-esteem and anxiety and its components. The relationship between self-esteem and anxiety was investigated in several research studies. However, the dependency of these two psychological factors was rarely investigated. Statistical methods are appropriate tools for analyzing the dependency structure between psychological factors. Considering this fact, copula functions are the most suitable tools for this purpose. Copula functions have been widely used for modeling in the fields of hydrology, meteorology and environmental processes. To the best of the authors’ knowledge, this is the first time that these functions have been used in psychological analysis. In this regard, the self-esteem and anxiety of a number of undergraduate students in Iran was measured. Then, different copula functions were fitted to the joint self-esteem and anxiety data and the best copula function was selected. Subsequently, based on the procedure proposed by Dehghani et al. (2019) [26] and Dehghani et al. (2020) [27], the conditional probabilities of self-esteem and anxiety were extracted.

## 2. Material and Methods

### 2.1. Data Collection

The present study was conducted to determine the dimensions of self-esteem of students at the Vali-e-Asr University of Rafsanjan. The population of this university included 6523 undergraduate students, of which 200 students were selected randomly based on a proportional stratified sampling method from the faculties of engineering, literature and humanities, Iranology, administrative sciences and economics, sciences, mathematics and agriculture. The number of students selected in each faculty was proportional to the population of each faculty. Therefore, 55, 34, 10, 14, 30, 14, 43 and 10 people were selected from the engineering, literature and humanities, Iranology, administrative sciences and economics, sciences, mathematics and agriculture faculties. Along with the questionnaire, a letter was sent to all potential respondents stating the subject of the research and asking them to answer the questionnaire if they were satisfied. It was also announced to all respondents that the results of this research will be published. All respondents were asked to refrain from mentioning their names in the replies, and how to answer the questions was clearly explained to them. Respondents were also assured that their answers would be kept confidential by the researcher. After collecting the questionnaires and checking them, the questionnaires that were not fully answered were excluded from the study and totally, 141 questionnaires were analyzed. Data collection tools include the Coopersmith Self-esteem Inventory (SEI), the Demographic Information Questionnaire and the Zang Anxiety Scale. Overall, 42% of the respondents were male and 58% were female.

### 2.2. Coopersmith Self-Esteem Inventory (SEI)

One of the most common scales to assess self-esteem is the Coopersmith self-esteem questionnaire. The school form of this scale is based on an extensive review of the fundamentals, implications, and inter-relationships of self-esteem. Most of the materials included in the scale are the adjusted materials of the Rogers and Diamond (1954) [28] scale, but other materials have been added. All sentences are arranged in a way that can be used by children from 10 years and up. This scale consists of 8 items whose content is so clearly positive or negative that the respondent can easily choose the best option related to him/her. The questionnaire is accessible via the following link: https://fetzer.org/sites/default/files/images/stories/pdf/selfmeasures/Self_Measures_for_Self-Esteem_COOPERSMITH_SELF-ESTEEM_INVENTORY.pdf (accessed on 4 April 2021). It must be noted that a Persian translation of this questionnaire was used, which was also widely used in previous research studies in Iran. The score on this scale should be evaluated as an indicator of defensive feedback to the test rather than as a sign of falsehood. Thus, the self-esteem scale has been developed to measure self-feedback in the social, family, school, and personal domains, and a false scale has been added [29]. The self-esteem questionnaire consists of 58 items that describe a person’s feelings, beliefs, or reactions. The method of scoring this test is zero and one, which means that in some questions the answer “yes” produces the one score and the answer “no” produces a value of zero. The rest of the questions are scored in reverse. The score of the false scale is not calculated as a total of the scores. Based on the summation of scores, any score less than 26, between 27 and 43, and of 44 and more is ranked poor, average and strong self-esteem, respectively.

Zang Self-Rating Anxiety Scale: The Zang Self-Rating Anxiety Scale (SAS) was developed by William Zang (1971) [30]. This scale is widely used to measure general anxiety and is based on the physical-emotional symptoms of anxiety. In order to create this questionnaire, diagnostic criteria have been used that are consistent with the most common characteristics of anxiety. In this way, the clinical interviews of anxious clients were recorded in detail and, later, each of the cases were used in the making of the test. This scale includes 20 items. The range of scores is between 20 and 80. People with less anxiety obtain lower scores and people with more anxiety receive higher scores on this scale. Scores between 20 and 44 show normal anxiety; 45 to 59, mild anxiety; 60 to 74, severe anxiety; and 75 and above present very severe anxiety. This questionnaire is accessible via the following link: https://psychology-tools.com/test/zung-anxiety-scale (accessed on 4 April 2021). In addition, it is worth mentioning that for the SEI questionnaire, a Persian translation of the SAS was used.

### 2.3. Copula Functions

For modeling of the bivariate distributions, the dependency between the marginal values must be considered. The copula is capable of analyzing the dependency structure of bivariate distributions. A two-dimensional copula is a distribution function on [0, 1] × [0, 1], with standard uniform marginal distributions. Archimedean copula functions are widely used in different studies, and are defined as:

(1) $$C_{\phi_{\theta }}\left(u_{1},u_{2}\right)=\phi_{\theta }^{-1}\left(\phi_{\theta }\left(u_{1}\right)+\phi_{\theta }\left(u_{2}\right)\right)$$

where $\varphi_{\theta }$ is a generator of the copula and $\phi_{\theta }:\left[0,\;1\right]\to \left[0,\;\;\infty \right)$ is a mapping which satisfies the following conditions, according to [31].

(2) $$\varphi_{\theta }\left(1\right)=0,\;{\left(-1\right)}^{i}\frac{d^{i}}{dt^{i}}\varphi_{\theta }^{-1}\left(t\right)>0,\;i=1,2$$

Five Archimedean copula functions, including those of Clayton, Frank, Gumbel–Hougaard, Joe and Ali–Mikhail–Hag, and two copula functions, including the Normal and Plackett versions, were used for modeling the dependency structure of self-esteem and anxiety. The properties of these functions are presented in Table 1.

To choose the best copula function among those presented above, it is necessary to use a goodness of fit (GOF) test. For this purpose, the GOF test proposed in [31,32,33] was utilized.

Based on the dependency between self-esteem and anxiety, it is possible to model this dependency according to copula and the conditional probability can be calculated between their different classes. Suppose that self-esteem and anxiety are presented by *X* and *Y*, respectively. So, the conditional cumulative distribution function of *X* given *Y* = *y* can be calculated as follows according to [26].

(3) $$\begin{array}{cc} F_{X|Y}\left(a|y\right) & =P\left(X\right)\leq a|Y=y\\ & =\int_{-\infty }^{a}f_{X|Y}\left(x|y\right)dx=\frac{1}{g\left(y\right)}\int_{-\infty }^{a}f_{X,Y}\left(x,y\right)dx\\ & =\int_{0}^{F\left(a\right)}\frac{\partial^{2}}{\partial u_{1}\partial u_{2}}du\;\;\;\;\;\;\;\;\;\mathrm{by}\;\mathrm{letting}\;F\left(x\right)=u_{1}\;\mathrm{and}\;G\left(y\right)=u_{2}\\ & =\int_{0}^{F\left(a\right)}c\left(u_{1},u_{2}\right)du \end{array}$$

where $c\left(u_{1},u_{2}\right)$ is the copula and *f(x)* and *g(y)* are the probability distribution functions and *F* and *G* are the cumulative distribution functions.

In the next step, it is possible to calculate conditional probability of the self-esteem class given the anxiety class as follows:

(4) $$P\left(a<X<b|c<Y<d\right)=\frac{\int_{F\left(a\right)}^{F\left(b\right)}\int_{G\left(c\right)}^{G\left(d\right)}c\left(u_{1},u_{2}\right)dvdu\;}{G\left(d\right)-G\left(c\right)}$$

To select the best cumulative distribution function for the self-esteem and anxiety data and determine F(x) and G(y), five distributions, including normal, Inverse Gaussian, gamma, generalized pareto and log-normal, were utilized as presented in Table 2. The whole procedure was conducted using R software.

## 3. Results

In this study, the dependence structure between self-esteem and anxiety in university students was investigated using a novel approach. For this purpose, copula functions were used. The results of 141 questionnaires were used for this analysis. First, five probability distribution functions were fitted to the anxiety and self-esteem data to find the best marginal value. The null hypothesis is $H_{0}:X\sim F\left(x\right)$, where F(x) could be one of the distribution functions in Table 2. The Kolmogrov–Smirnov test was used to confirm that the samples follow the distribution functions in Table 2. The results are presented in Table 3. Based on the results, for self-esteem, all five probability distributions were fitted to the data satisfactorily. Overall, the Inverse Gaussian distribution had the best fit according to the AIC. In addition, for anxiety, all probability distributions were fitted, while gamma had the best fit.

In the next step, the copula functions were fitted to the joint self-esteem and anxiety data. The results are presented in Table 4.

**Figure 1:**
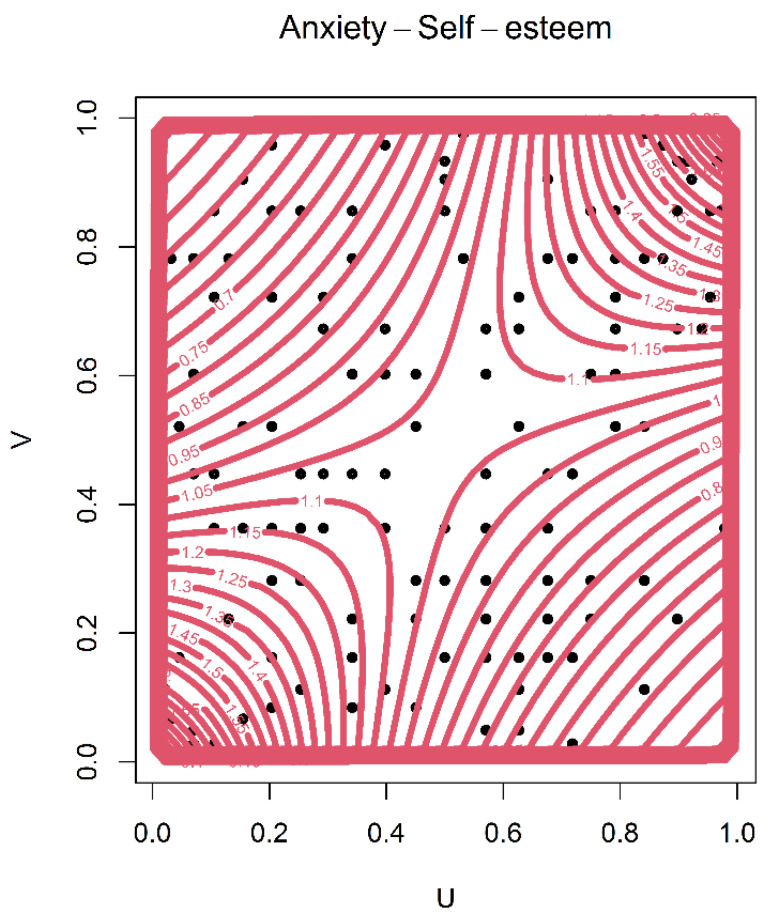
The contour plot of the Frank copula with scatter plot of the joint anxiety and the self-esteem combinations.

**Figure 2:**
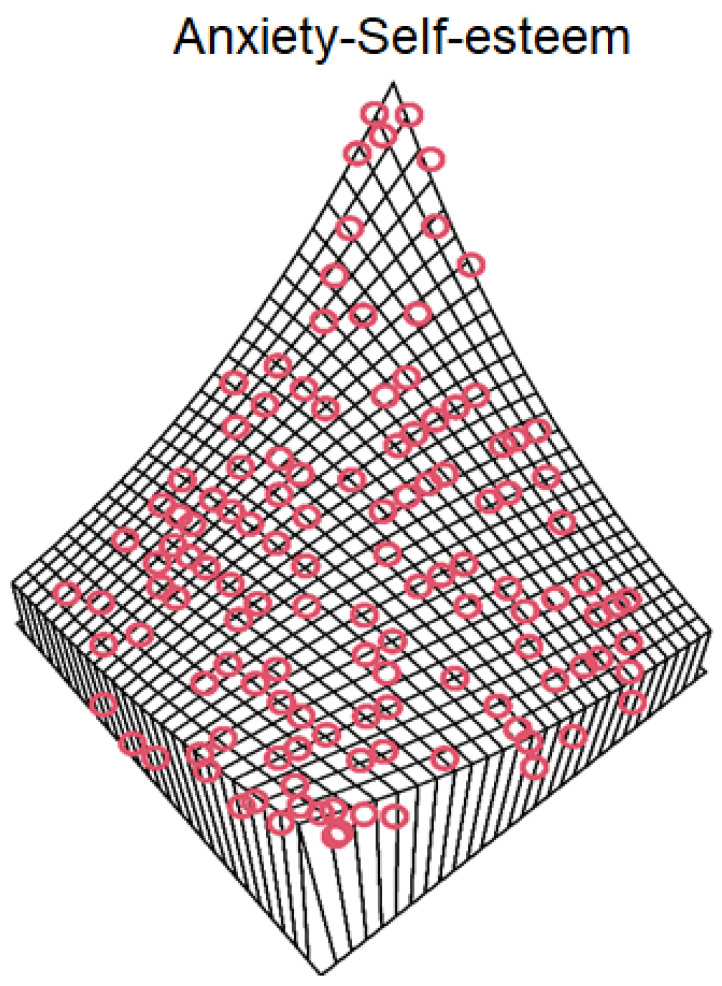
The perspective plot of the Frank copula and the scatter plot of the joint anxiety and self-esteem combinations.

The critical value and *p*-value were computed using 1000 iterations. According to the results, all copula functions were fitted to the joint self-esteem and anxiety data based on the *p*-values. The confidence interval of the copula parameter was estimated to be between 2.5% and 97.5%. It is noted that a narrow bandwidth exists for θ in all copula functions, which shows the robustness of the modeling. However, for a robust selection, the tail dependence of the data was evaluated using the copula package in R software. Based on this evaluation, the data has neither lower nor upper tail dependence. It must be noted that the Frank, Normal, Plackett and Ali–Mikhail–Hag functions do not allow for tail dependence. Among these copula functions, Frank had the best fit. The contour plot and perspective plot of the Frank copula and the scatter plot of the data are plotted in Figure 1. In addition, the perspective plot of the Frank copula and the scatter plot of the joint data are plotted in Figure 2. It is obvious that the data follows the Frank copula appropriately. Therefore, it was selected to model the dependence structure of self-esteem and anxiety and to prepare a probability matrix based on their classes.

In the final step, the conditional probabilities of self-esteem and anxiety were calculated according to Equation (4) and the Frank copula. The probability matrix between self-esteem and anxiety was extracted and is presented in Table 5.

According to Table 5, in the case of poor self-esteem, severe or very severe anxiety is is expected. Probabilities of 28% and 70.4% were assigned to severe and very severe anxiety, respectively, when self-esteem was in in the poor class. This means that with more than 98% probability, the university students with poor self-esteem, in this research, suffered from severe or very severe anxiety. A very negligible probability was assigned to normal and mild anxiety. However, when the self-esteem was in the average class, the results were completely different. A very negligible probability was assigned to the normal condition. This means that even average self-esteem cannot lead to normal anxiety. Overall, 36%, 17.9% and 46% probabilities were assigned to the mild, severe and very severe anxiety classifications. This shows that about half of the university students with average self-esteem may experience very severe anxiety. Finally, strong self-esteem may lead to normal and mild anxiety, with a high probability. Probabilities of 44.3% and 35.6% were allocated to the normal and mild anxiety classifications, respectively, and 19.8% to severe anxiety. A probability of just 0.33% was allocated to very severe anxiety.

## 4. Conclusions

In this study, a new approach was introduced for psychological analysis using copula functions. To construct the dependency structure and produce the probability matrix of two dependent variables, the self-esteem and anxiety of university students in Iran were considered. Based on the results, the dependency between these variables can be modeled using the Frank copula. The probability matrix showed that poor self-esteem leads to severe and especially very severe anxiety, with a very high probability and a negligible probability allocated to mild and normal anxiety. Conversely, strong self-esteem is associated with normal and mild anxiety, with about 80% probability. However, about 20% probability is allocated to severe anxiety, which shows that even strong self-esteem may be associated with severe anxiety. The average class of self-esteem is associated with different behaviors than the poor and strong classes. The highest probability belongs to very severe anxiety, followed by mild anxiety. This study proposed the application of copula in psychological studies. Generally, copulas are suitable for considering the dependence structure between two or more variables. In psychology, different combinations of variables, with high or low correlation, could be studied. However, correlation does not show dependency. Instead, copula functions are the means of analyzing dependency. In copula applications, the high or low correlation between the variables is not important and some copula functions cover a wide range of correlations, while others are developed for variables with high or low correlation. Therefore, it is possible to use these functions in a wide range of psychological studies.

## Figures and Tables

**Table 1:** Copula functions used in this study.

Copula	Domain of Dependence Parameter θ	Copula Function C(u,v)	Kendall’s Tau (τ)
Clayton	[−1, ∞)\{0}	${\left\{max\left(u_{1}{}^{-\theta }+u_{2}{}^{-\theta }-1,0\right)\right\}}^{\frac{-1}{\theta }}$	[−1, 1]
Frank	(−∞, ∞)\{0}	$-\frac{1}{\theta }log\left[1+\frac{\left\{\exp\left(-\theta u_{1}\right)-1\right\}\left\{\exp\left(-\theta u_{2}\right)-1\right\}}{exp\left(-\theta \right)-1}\right]$	[−1, 1]
Gumbel–Hougaard	[1, ∞)	$exp\left[-{\left\{{\left(-logu_{1}\right)}^{\frac{1}{\theta }}+{\left(-logu_{2}\right)}^{\frac{1}{\theta }}\right\}}^{\theta }\right]$	[0, 1]
Joe	[1, ∞)	$1-{\left\{{\left(1-u_{1}\right)}^{\theta }+{\left(1-u_{2}\right)}^{\theta }-{\left(1-u_{1}\right)}^{\theta }{\left(1-u_{2}\right)}^{\theta }\right\}}^{\frac{1}{\theta }}$	[0, 1]
Ali–Mikhail–Hag	[−1, 1]	$\frac{u_{1}u_{2}}{1-\theta \left(1-u_{1}\right)\left(1-u_{2}\right)}$	[−0.18, 0.33]
Normal	[−1, 1]	$\int_{-\infty }^{\Phi^{-1\left(u_{1}\right)}}\int_{-\infty }^{\Phi^{-1\left(u_{1}\right)}}\frac{1}{2\pi \sqrt{1-\theta^{2}}}exp\left\{\frac{2\theta st-s^{2}-t^{2}}{2\left(1-\theta^{2}\right)}\right\}dsdt$	[−1, 1]
Plackett	[0, ∞)	$\frac{\left\{1+\left(\theta -1\right)\left(u_{1}+u_{2}\right)\right\}-\sqrt{{\left\{1+\left(\theta -1\right)\left(u_{1}+u_{2}\right)\right\}}^{2}-4u_{1}u_{2}\theta \left(\theta -1\right)}}{2\left(\theta -1\right)}$	[−1, 1]

**Table 2:** The distribution functions used in this study.

Distribution	CDF
Normal	$F\left(x\right)=\Phi \left(\frac{x-\mu }{\sigma }\right)$ 0<x<∞ Φ is the CDF of standard normal distribution
Log-normal	$F\left(x\right)=\Phi \left(\frac{\ln\left(x\right)-\mu }{\sigma }\right)$ 0<x<∞
Inverse Gaussian	$F\left(x\right)=\Phi \left(\sqrt{\frac{\lambda }{x}}\left(\frac{x}{\mu }-1\right)\right)+\Phi \left(-\sqrt{\frac{\lambda }{x}}\left(\frac{x}{\mu }+1\right)\right)e^{\frac{2\lambda }{\mu }}$ 0<x<∞ Φ is the CDF of standard normal distribution
Generalized Pareto	$F\left(x\right)=1-{\left(1+k\frac{\left(x-\mu \right)}{\sigma }\right)}^{\frac{-1}{k}}$ μ≤x<∞
Gamma	$F\left(x\right)=\frac{\Gamma_{\frac{x}{\beta }\left(\alpha \right)}}{\Gamma \left(\alpha \right)}$ 0<x<∞

**Table 3:** Estimated parameters of marginal distributions fitted to the self-esteem and anxiety data.

	Margin	Parameters	AIC	K–S Test	
				Statistics	*p*-Value
Self-esteem	**Normal**	** (μ, σ) = (26.77, 4.81)**	853.42	0.065	0.56
	Log-normal	(μ, σ) = (3.27, 0.19)	853.2	0.08	0.32
	Inverse Gaussian	(λ, μ) = (828.53, 26.77)	**848.2**	0.06	0.67
	Gamma	(α, β) = (30.95, 0.87)	849.4	0.07	0.47
	Generalized pareto	(μ, σ, k) = (18.47, 16.88, −1.03)	857.2	0.082	0.28
Anxiety	Normal	(μ, σ) = (46, 9.81)	1039.1	0.066	0.55
	Log-normal	(μ, σ) = (3.81, 0.21)	1038.54	0.062	0.62
	Inverse Gaussian	(λ, μ) = (1012.1, 46)	1040.2	0.07	0.47
	Gamma	(α, β) = (22, 2.09)	**1037.4**	0.048	0.87
	Generalized pareto	(μ, σ, k) = (31.32, 24.54, −0.67)	1042.3	0.076	0.36

**Table 4:** Results of goodness of fit of copula functions.

Copula	θ	Test Statistic	*p*-Value
Clayton	0.469	0.962	0.19
Frank	1.734	0.717	0.58
Gumbel–Hougaard	1.236	0.752	0.48
Joe	1.343	1.134	0.07
Ali–Mikhail–Hag	0.593	0.917	0.22
Normal	0.305	0.728	0.53
Plackett	2.386	0.729	0.52

**Table 5:** The probability matrix between self-esteem and anxiety combinations. The values are in percent.

			Anxiety			
			Normal	Mild	Severe	Very Severe
		Number of Students	67	62	11	1
**Self-esteem**	Poor	67	1.33	0.4	28	70.4
	Average	73	0.32	36	17.9	46
	Strong	1	44.3	35.6	19.8	0.33

## Data Availability

The data are available from the first author on reasonable request.
